# Design of Chinese traditional Jiaoyi (Folding chair) based on Kansei Engineering and CNN-GRU-attention

**DOI:** 10.3389/fnins.2025.1591410

**Published:** 2025-05-21

**Authors:** Xinyan Yang, Nan Zhang, Jiufang Lv

**Affiliations:** ^1^College of Furnishings and Industrial Design, Nanjing Forestry University, Nanjing, China; ^2^School of Design Art and Media, Nanjing University of Science and Technology, Nanjing, China; ^3^Co-Innovation Center of Efficient Processing and Utilization of Forest Resources, Nanjing Forestry University, Nanjing, Jiangsu, China; ^4^NJFU Academy of Chinese Ecological Progress and Forestry Development Studies, Nanjing, Jiangsu, China

**Keywords:** Kansei Engineering, affective cognition, deep learning, Jiaoyi chair design, user preference prediction

## Abstract

**Backgrounds:**

This study innovatively enhances personalized emotional responses and user experience quality in traditional Chinese folding armchair (Jiaoyi chair) design through an interdisciplinary methodology.

**Goal:**

To systematically extract user emotional characteristics, we developed a hybrid research framework integrating web-behavior data mining.

**Methods:**

1) the KJ method combined with semantic crawlers extracts emotional descriptors from multi-source social data; 2) expert evaluation and fuzzy comprehensive assessment reduce feature dimensionality; 3) random forest and K-prototype clustering identify three core emotional preference factors: “Flexible Refinement,” “Uncompromising Quality,” and “ergonomic stability.”

**Discussion:**

A CNN-GRU-Attention hybrid deep learning model was constructed, incorporating dynamic convolutional kernels and gated residual connections to address feature degradation in long-term semantic sequences. Experimental validation demonstrated the superior performance of our model in three chair design preference prediction tasks (RMSE = 0.038953, 0.066123, 0.0069777), outperforming benchmarks (CNN, SVM, LSTM). Based on the top-ranked preference encoding, we designed a new Jiaoyi chair prototype, achieving significantly reduced prediction errors in final user testing (RMSE = 0.0034127, 0.0026915, 0.0035955).

**Conclusion:**

This research establishes a quantifiable intelligent design paradigm for modernizing cultural heritage through computational design.

## 1 Introduction

In today’s design and manufacturing fields, consumers’ demands for products have surpassed basic functionality, placing greater emphasis on the emotional experience and personalized satisfaction they provide. As an indispensable part of daily life, the design of armchairs must not only meet ergonomic requirements but also align with users’ emotional cognition and aesthetic needs (Riley et al., 2021). This paper aims to explore the integration of Kansei Engineering and deep learning techniques in the design of armchair shapes, in order to precisely capture users’ emotional needs and optimize product design.

Kansei Engineering, an interdisciplinary field combining psychology, engineering, and cognitive science, is dedicated to converting consumers’ emotional needs and subjective feelings into quantifiable design elements. By studying consumers’ emotional and sensory responses, it provides more scientific theoretical support for product design. In armchair shape design, Kansei Engineering can assist designers in deeply understanding users’ emotional cognition and reactions, thereby creating products that better meet users’ expectations. Mao, YC employed eye-tracking technology to investigate the relationship between the design styles, aesthetic performances, and visual attention of four types of Chinese-style chairs (Mao, 2024). The results revealed that both materials and decorations exert independent as well as interactive effects. Traditional Chinese patterns enhanced the design styles and aesthetic performances, particularly when applied to wooden chairs. Except for wooden chairs with traditional patterns on the backrests, there were significant differences in visual attention among the chair designs (pp. 220–232). Zheng, WS conducted a study on preference intentions for chair designs in the Suzhou style of the Ming Dynasty. By summarizing aspects such as materials, decorative materials, shapes, structures, and cultural connotations of Suzhou-style chairs, the differences between Suzhou-style chairs of the Ming and Qing dynasties were analyzed (pp. 724–727). There is relatively limited research on traditional Chinese chairs, and the aforementioned two articles did not address the influence of chair contours on user preferences. However, there is abundant research on product form and user preferences. For instance, Nanyi Wang utilized the SSA-LSTM-Attention model, combined with Kansei Engineering, to explore the nonlinear relationship between user emotions and design features of intelligent driving cockpits in new energy vehicles (NEVICs), demonstrating higher prediction accuracy. Additionally, Nanyi Wang employed electroencephalography (EEG) and eye-tracking (ET) technologies to investigate the impact of different ESR bionic designs (animal-shaped, human-shaped, and abstract-shaped) on users’ emotional preferences. Since research on traditional Chinese furniture predominantly adopts a cultural perspective and lacks the utilization of advanced technologies such as computer science and eye-tracking, this study attempts to conduct a quantitative investigation into the traditional cultural form symbols and aesthetic aspects of folding chairs by integrating advanced technologies (Wang and Xu, 2024).

Emotional feature extraction serves as a crucial bridge between user emotions and product design. In the emotional feature extraction section of this paper, various methods such as the KJ method, K-prototype clustering, triangular fuzzy numbers, and Random Forest are introduced. The KJ method is a technique for forming systematic thoughts and insights by categorizing and organizing large amounts of information, aiding in extracting key emotional vocabulary from user research. K-prototype clustering is a clustering algorithm that combines numerical attributes and categorical attributes, suitable for processing mixed-type information in user evaluation data. Triangular fuzzy numbers offer a method for handling uncertain information, enabling more accurate quantification of users’ vague emotional needs. Random Forest, as an ensemble learning method, improves the accuracy and stability of classification and regression tasks by constructing multiple decision trees.

The Random Forest (RF) algorithm has demonstrated significant application value in design optimization, behavior prediction, and performance evaluation across multiple fields. Shi and Zhang (2014) proposed an emotional design method that successfully achieved dimensionality reduction of design elements and mapping of emotional needs by combining Random Forest regression and association rules, applying it to elevator design schemes (p. 1408–1409). Chen (2012) utilized Artificial Neural Networks (ANN), Random Forest decision trees, and Gradient Boosting Regression methods to construct a prediction model for consumers’ preferences toward technological products, providing data-driven decision support for product design. In product design and user psychological analysis (p. 023501). Lou et al. (2020) proposed a comprehensive decision-making method combining cloud models with electroencephalogram data, utilizing a Random Forest classifier to classify customers’ psychological states with an average accuracy of 90.76%, providing a scientific basis for product scheme evaluation (p. 101028). Wu et al. (2023) constructed an intelligent SSS standard system oriented by product categories through the Random Forest algorithm and Recursive Feature Elimination with Cross-Validation, achieving efficient screening and optimization of design features (p. 110586). In the field of image generation, Hayakawa et al. (2025) developed a machine learning framework based on ensemble learning and Shapley Additive exPlanations (SHAP), filtering training data through a Random Forest regression model to construct a tire tread image generation model, with experimental results showing superior performance compared to traditional multiple regression models (p. 22–51). Liu et al. (2024) further combined Particle Swarm Optimization (PSO) with the Random Forest algorithm to develop a mapping matrix connecting design features with sustainability factors, and optimized the number of branches and maximum depth of the RF algorithm through PSO, significantly enhancing the SAPAD-FQFD model’s ability to identify user needs and design functions (p. 2944–2955).

With the rapid development of deep learning techniques, their application in the field of product design has become increasingly widespread. In the deep learning section of this paper, the CNN-GRU-Attention model is highlighted, which combines Convolutional Neural Networks (CNN), Gated Recurrent Units (GRU), and the Attention mechanism, enabling efficient processing and analysis of image data to extract deep feature information. Currently, this model is widely used in various industry predictions. For example, Kang et al. (2025) employed CNN-GRU-Attention to extract natural biological information for creating diverse biomimetic design schemes (p. 126121).

**Figure 1:**
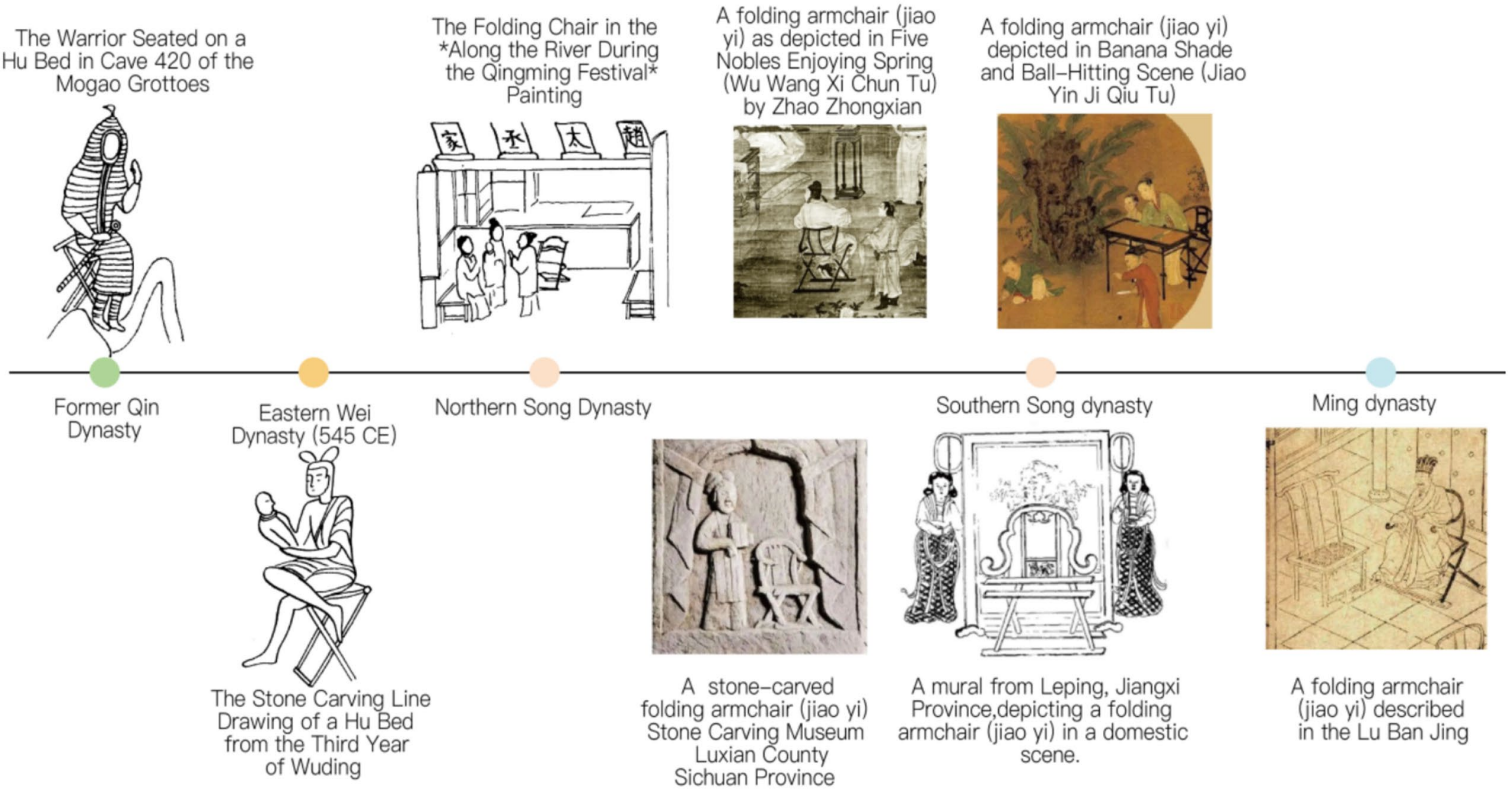
Development of the ‘The Jiaoyi’ (traditional Chinese folding armchair). Image source: Created by the author.

Based on the aforementioned literature research, this study conducts research to address the following questions: how to enhance personalized emotional responses and user experiences in the design of traditional Chinese folding armchairs (folding chairs); how to identify the core factors that influence users’ emotional preferences for folding chairs; how to design new folding chair prototypes that better meet users’ preferences; and how to establish a quantifiable intelligent design paradigm for the modernization of cultural heritage. The specific content of the article is arranged as follows: The first part is the Introduction (Kenny et al., 2024). The second part introduces relevant theories. The third part covers the research framework and process, as well as analysis and validation of design results. The fourth part is the Discussion, and the fifth part is the Conclusion. The paper aims to analyze armchair shape and pattern features, explore the design mechanism of armchair morphology, obtain a prediction model for armchair shape preferences, and investigate how to integrate armchair morphology mechanisms into modern furniture design to meet modern aesthetic demands (Figure 1).

## 2 Relevant theories

### 2.1 Chinese traditional folding armchair – the Jiaoyi

**Figure 2:**
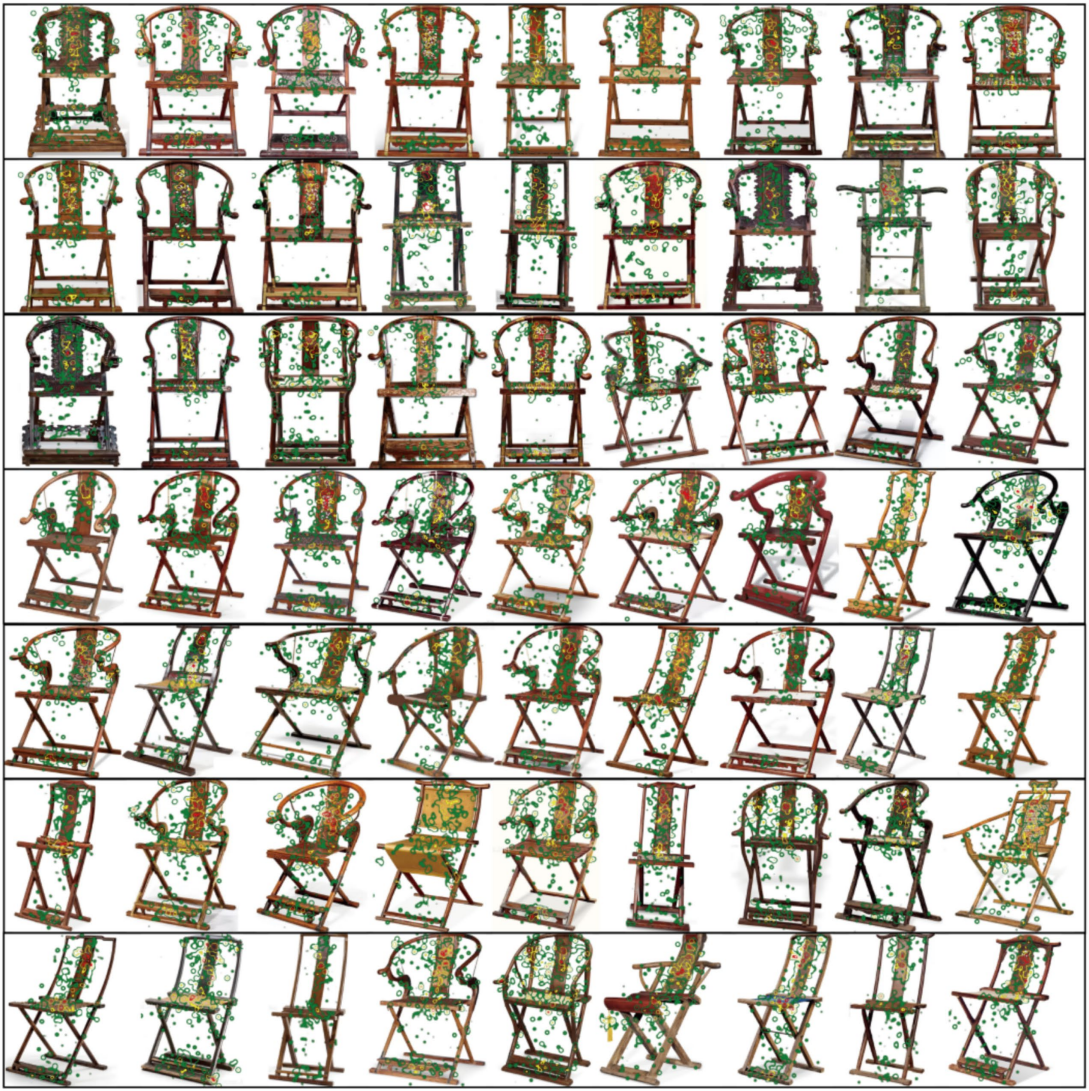
User visual sequence feature map. Image source: Created by the author.

The Jiaoyi, or Chinese traditional folding armchair, is a gem among Chinese traditional furniture, boasting a long history that can be traced back to the “Hu Chuang” (a type of folding stool) used by ancient northern ethnic minorities. Introduced during the late Eastern Han Dynasty and utilized within the imperial court during the Han Dynasty, the Hu Chuang originated from the northwest regions. During the former Qin Dynasty, the Hu Chuang resembled a “mazha” (a simple foldable stool), as first recorded in the “Book of the Later Han Dynasty: Chronicles of Five Elements.” The distinctive feature of the Hu Chuang was its crossed and slanted legs, a design that ensured stability when in use, differing from traditional four-legged upright beds. The introduction and adoption of the Hu Chuang reflected the influence of Hu people’s living customs on China against the backdrop of social unrest and mixed ethnic residence. The portability and versatility of the Hu Chuang made it an essential piece of furniture in social life at that time. During the Wei, Jin, Northern, and Southern Dynasties, the Hu Chuang was widely used on city walls, in outdoor courtyards, during military campaigns, and for hunting activities. Furthermore, the use of the Hu Chuang changed the sitting posture of the Chinese people, shifting from the traditional kneeling posture to the vertical leg-hanging posture, known as “ju” or “ju-sitting” at that time. Before the Han Dynasty, the Hu Chuang was in its infancy stage, while by the Song Dynasty, it had reached maturity and was renamed “Jiaoyi” (Figure 2).

### 2.2 Emotional cognition

The theory of emotional cognition emerged in the mid-20th century as a deepening of the cognitive branch of psychology. It explores the process through which individuals process external stimuli via the nervous system to generate emotional experiences, establishing a theoretical foundation for the interaction between emotion and cognition. In the field of product design, this theory has become a core guide for enhancing user experience and product value. In recent years, researchers have focused on the deep integration of emotional interaction and user cognition, utilizing multimodal measurement techniques and advanced algorithms to drive innovations in emotional design methodologies. Yang and Chen (2008) from the perspective of interaction design, emphasized the role of perceptual senses, cognitive experience, and behavioral reflection in deepening users’ emotional experiences (p. 51–54). Zhang (2009) analyzed the impact of aesthetic, semantic, and symbolic features of product design on cognitive and emotional responses, revealing the interactive mechanism between them in user behavior (p. 205–207). With technological advancements, Ding et al. (2021) proposed a color emotional design method based on eye movements and Event-Related Potentials (ERPs), constructing a cognitive framework for user emotional transmission and optimizing the design process (p. 871–889). Xi et al. (2016) ased on Kansei Engineering theory, utilized virtual reality technology to build an image description model for user emotional experiences, extracting key features. In research on consumer behavior and product perception (p. 98–109). Lin et al. (2019) explored the correlation between product shape elements and consumer emotional cognition through gray relational analysis and Kansei Engineering, providing a scientific basis for design optimization (p. 74–83).

Zhang et al. (2019) employed Kansei Engineering techniques to explore the relationship between product color and brand image, validating the effectiveness of their method through automotive color research (p. 130–136). Zhang et al. (2023) combined Electroencephalogram (EEG) and eye-tracking technology to reveal significant brain electrical responses during the user perception phase (p. 9577).

Furthermore, studied the classification of product modeling features and the mapping mechanism with users’ image needs through an emotional cognition model, helping designers better understand the matching relationship between user needs and modeling features. Li and Ye (2012) analyzed the psychological perception of modern furniture using morphological semantics, providing a quantitative solution for furniture design that aligns with user cognitive needs (p. 536–539). Zhong et al. (2018) constructed an information recommendation system based on an emotional cognition model, improving information classification and recommendation efficiency through quantitative coordinates (p. 133–146). In physiological experiments and emotional preference measurements, Guo et al. (2018) successfully identified subtle emotional responses of users to product appearances using Event-Related Potentials (ERP) technology (p. 299–308). Constructed user subconscious behavior trajectories through data mining, providing a guidance method for product design based on users’ unconscious behaviors. Wang and Zhou (2020) combined Interactive Genetic Algorithms with the Fuzzy Kano Model (FKM) to explore users’ emotional needs for product morphology, driving evolutionary design in product modeling (p. 102901). In the fields of industrial design and packaging analysis, Xiao and Cheng (2020) proposed a mapping method for industrial robot-perceived image space and design elements, validating its effectiveness through virtual reality and physiological experiments. Modi and Singh (2024) analyzed visual attention and emotional arousal in perfume bottle packaging design using eye-tracking technology, providing a basis for packaging optimization in an e-commerce environment (p. 82563–82588). Kang (2021) extracted Kansei vocabulary that most resonates emotionally with customers through factor analysis and fuzzy analytic hierarchy process, providing an efficient tool for product modeling design. In terms of intelligent generation and design evaluation, the integration of technologies such as Generative Adversarial Networks (GANs) and EEG experiments has achieved the integration and matching degree assessment of user emotional images in the intelligent design process. Colombo et al. (2016) has also contributed significantly to this field (p. 7–14).

In summary, the fusion of emotional cognition and multimodal measurement technologies provides scientific and precise methodological support for product design. By comprehensively utilizing techniques such as Kansei Engineering, physiological experiments, data mining, and intelligent generation, researchers can gain a deeper understanding of users’ emotional needs, optimize product design processes, and ultimately enhance user experience and market competitiveness.

### 2.3 Emotion feature extraction techniques

#### 2.3.1 KJ Method

The KJ Method, also known as the Kawakita Jiro Method, is a qualitative data analysis technique proposed by Japanese cultural anthropologist Jiro Kawakita in the 1960s. The core of this method lies in synthesizing and structuring large volumes of unstructured information, with the aim of distilling consensus or identifying the core of intricate problems, thereby facilitating creative problem-solving. The essence of this method is card sorting and the construction of affinity diagrams. It leverages human intuition and collaborative strategies, such as brainstorming and affinity grouping, to integrate information and uncover hidden patterns. Recognized as a powerful tool for collective decision-making, it emphasizes the importance of collective intelligence and the systematic organization of fragmented data (Liu et al., 2021; Wang and Xu, 2024).

In our research on folding chair design, we have chosen the KJ Method to collect and organize emotional cognitive vocabulary related to chair design, as this method aligns with our research objectives. Firstly, the KJ Method provides a structured yet flexible framework for handling the diverse and often disparate emotional responses that individuals may have toward chair design. Through card sorting, we can break down complex emotional experiences into manageable components, systematically categorize and analyze these responses, and gain a more comprehensive understanding of users’ preferences and emotional needs (Lei et al., 2024). Additionally, the KJ Method emphasizes visual representation through affinity diagrams, which facilitates clear communication and consensus-building among team members. This visual aid not only helps in organizing the data but also serves as a reference point for further analysis and discussion, ensuring that all team members are on the same page throughout the research process (Zhu and Lv, 2023).

In terms of implementation, we will first gather a large amount of emotional cognitive vocabulary related to folding chair design from various sources, such as user interviews, online reviews, and design literature. These vocabulary terms will then be written on individual cards. Subsequently, we will conduct collaborative card sorting, where team members will group the cards based on perceived similarities and relationships. This process will be iterative, allowing for adjustments and refinements as new insights emerge. Finally, we will create an affinity diagram to visually represent the grouped vocabulary, highlighting the key themes and patterns that emerge from the data.

#### 2.3.2 Kansei Engineering

Kansei Engineering, originating in Japan during the 1970s, is an engineering methodology designed to systematically translate users’ emotional needs (Kansei) into tangible technical parameters for product design. Central to this approach is the quantification of users’ affective responses (e.g., emotions, preferences, perceptions) to establish a mapping relationship between subjective user experiences and product design elements. To achieve this, Kansei Engineering adopts an interdisciplinary approach, integrating techniques from statistics, psychology, and engineering. Data on users’ emotional evaluations of products are collected through tools such as questionnaires and the Semantic Differential Method (SDM). Statistical models, including Factor Analysis and Regression Analysis, are then applied to decode correlations between design variables (e.g., dimensions, materials, colors) and user emotions. By bridging subjective emotional demands with actionable design specifications, Kansei Engineering provides scientifically grounded decision-making frameworks for product development. This methodology enables precise transformation of user-centric emotional requirements into technical solutions, thereby enhancing the emotional value and user experience of products.

In our research on folding chair design, Kansei Engineering serves to bridge the gap between subjective emotional needs and actionable design specifications, thereby providing a scientifically grounded decision-making framework for product development. By precisely translating user-centric emotional requirements into technical solutions, Kansei Engineering enhances the emotional value and user experience of products, which is of paramount importance in the highly competitive furniture market. The advantages of utilizing Kansei Engineering are multifaceted. Firstly, it allows us to systematically collect and analyze data on users’ emotional evaluations of folding chairs through tools such as questionnaires and the Semantic Differential Method (SDM). This data-driven approach ensures that our design decisions are based on empirical evidence rather than intuition or guesswork. Secondly, by applying statistical models such as Factor Analysis and Regression Analysis, we can decipher the correlations between design variables (e.g., dimensions, materials, colors, patterns) and user emotions. This enables us to identify the key design elements that significantly influence users’ emotional responses and prioritize them in the design process (Fu et al., 2024b).

#### 2.3.3 Product morphological decomposition

Product Morphological Decomposition (Morphological Analysis) is a qualitative analysis method widely used in Kansei Engineering (KE) research. Originally proposed in 1948 by Fritz Zwicky, an astrophysicist at the California Institute of Technology, this approach systematically deconstructs and analyzes product morphology to reveal its intrinsic logic and structural principles, thereby providing theoretical foundations and inspiration for innovative design (Fu et al., 2024a). Key procedural steps include target product selection, morphological decomposition, analysis of form-function relationships, morphological feature extraction, and morphological reconstruction. For instance, Zhao et al. deconstructed the contour of a whiskey bottle into five morphological categories (cap, neck, body, posture, and base) to optimize the design process. In recent years, scholars have integrated computational aesthetics into morphological analysis, employing parametric techniques for enhanced precision. For example, Gunpinar et al. (2019) utilized modified Bézier curves to decompose automotive forms into nine core characteristic lines, significantly improving design accuracy (p. 683–723).

Research on the relationship between user preferences and product form spans multiple industries including automotive design, food packaging, furniture, and electronics. However, existing studies predominantly focus on ontological analysis of morphological elements (e.g., lines, proportions, materials) while failing to establish a comprehensive explanatory model that integrates “morphological features-user cognition-emotional feedback” mechanisms. From a semiotic perspective, the formal configuration of a cross-legged chair serves as materialized carriers of cultural codes, where the curvature of its backrest not only addresses ergonomic requirements but also metaphorically embodies the traditional philosophical concept of “harmony between humanity and nature.”

As a cultural symbol, the morphological characteristics of this chair type undergo semantic shifts across different cultural contexts. In Southeast Asian markets, the circular backrest is interpreted as a symbol of “family unity,” whereas in Nordic regions it is perceived as an expression of “minimalist aesthetics.” Such cross-cultural decoding discrepancies necessitate the development of context-sensitive semantic analysis frameworks to prevent cultural misinterpretations that may lead to market failures. Without incorporating correlational studies on user cognitive schemas (e.g., collective memory archetypes, cultural symbolic representations), morphological design risks descending into formalist self-expression.

This study specifically targets the Chinese market, where users demonstrate stronger preferences for cultural narratives embodied in traditional mortise-and-tenon structures. The research emphasizes that effective design solutions must consider both universal ergonomic principles and culture-specific symbolic systems, particularly the Chinese aesthetic tradition that values craftsmanship heritage and philosophical symbolism in material forms. By establishing a dynamic mapping between morphological parameters and contextualized user preferences, the study aims to construct a predictive model that balances cultural authenticity with contemporary functional requirements.

### 2.4 Emotion recognition and classification technology

Emotion Recognition and Classification hold significant theoretical and practical value in understanding human emotions, optimizing Human-Computer Interaction (HCI), and driving intelligent applications. In Social Media Analytics, emotion recognition technology helps enterprises accurately capture user feedback and opinions on products, providing data-driven decision support for product iteration and market strategy optimization. In Intelligent Customer Service Systems, the emotion classification function enables robots to more accurately identify users’ emotional states, thus providing personalized and contextualized service experiences (Chen and Bian, 2024). In Mental Health Monitoring, emotion recognition technology can be used for early detection and warning of potential psychological issues, providing intelligent support for individual mental health (Zheng, 2016).

With the rapid development of Deep Learning and Machine Learning technologies, emotion recognition and classification methods have evolved from rule-based methods to statistical learning and then to deep learning models, significantly improving accuracy and robustness. However, facing the diversity of emotional expression and cultural background differences, emotion recognition and classification still face numerous challenges, such as cross-cultural emotion variability and emotional ambiguity in complex contexts, which require further research and breakthroughs.

#### 2.4.1 Random Forest

Random Forest improves model prediction performance by combining multiple decision trees. Its core idea is to introduce diversity through Bootstrap sampling or random feature subset selection, ultimately integrating results through voting (for classification) or averaging (for regression). Bootstrap Aggregating (Bagging) sampling involves randomly drawing multiple subsets with replacement from the original dataset, with each subset independently training a decision tree (Dawar et al., 2019). This sampling approach helps increase model diversity and reduce overfitting. Random feature selection involves randomly selecting a subset of features when constructing each node of a decision tree and further selecting the optimal feature for splitting. In terms of classification prediction, Duong et al. (2025) used a Random Forest model to extract product attributes from customer reviews, accurately identifying reviews with high return risks (p. 109499). Utilized a Random Forest model to predict the yield strength of 540 casting alloys, demonstrating significantly better prediction accuracy across different strength ranges compared to traditional methods through classification analysis. Additionally, Valecha et al. (2018) proposed a time-evolving Random Forest classifier that successfully predicted consumer behavior through unique feature engineering, significantly improving the accuracy of product selection (p. 1–6). Mahajan et al. (2024) developed a Random Forest model optimized based on a genetic algorithm for predicting bending characteristics, with experiments showing that its target loss was significantly lower than that of Bayesian optimization methods, providing an efficient solution for complex performance predictions (p. 10700–10710).

In chair design research, we chose to employ the Random Forest algorithm to conduct feature weight analysis on the remaining vocabulary, with the aim of identifying keywords that have a significant impact on user preferences. Moreover, the ensemble nature of Random Forest enhances its predictive accuracy and stability, making it a reliable tool for feature selection and importance ranking. In terms of implementation, we will first preprocess the vocabulary data, including tokenization, stop-word removal, and stemming, to ensure that the data is in a suitable format for analysis. We will then apply the Random Forest algorithm to the preprocessed data, using user preference ratings as the target variable. By training the model on a subset of the data and evaluating its performance on a separate test set, we can assess the model’s predictive accuracy and the significance of each feature. Finally, we will extract the keywords with the highest feature weights, which will serve as the basis for subsequent data processing and model construction. These keywords not only represent the focal points of user attention regarding chair morphological characteristics but also provide valuable insights for designing chairs that better meet user preferences.

Specific steps are as follows:

Step 1: Data Preparation:

Input dataset $D=\{(x_{1},y_{1}),(x_{2},y_{2}),\ldots ,(x_{n},y_{n})\}$ , where $x_{i}$ represents the feature vector and $y_{i}$ represents the target variable (classification label or continuous value).

Set hyperparameters, including the number of trees (typically defaulted to 100), the maximum number of features (the number of features randomly selected for splitting in each tree), the maximum depth of the tree (controlling the complexity of the tree), and the minimum samples split (the minimum number of samples required for a node to split).

Step 2: Construct a Single Decision Tree.

1. Bootstrap Sampling:

Randomly drawn samples with replacement from the original dataset (allowing duplicates) to form the training subset $D_{\text{boot}}$ for that particular tree. The samples not drawn are referred to as Out-of-Bag (OOB) data, which can be used to evaluate model performance.

1. Recursive Node Initiation and Operations:

Recursively perform operations (including randomly selecting a subset of features, choosing the optimal split point, and splitting the node) starting from the root node until a stopping condition is met (such as reaching the maximum depth or having too few samples in a node).

Step 3: Ensemble Multiple Decision Trees:

Repeat Step 2 to construct n_estimators decision trees, forming the forest $\left\{T_{1},T_{2},T_{k.}\right\}$

Step 4: Prediction and Ensemble:

For classification tasks: For an input sample x, each tree outputs a class label, and the final result is determined by majority voting among all trees. As in Equation 1:


(1) $$\hat{y}=\text{mode}(\{T_{1}(x),T_{2}(x),\ldots ,T_{k}(x)\})$$

Regression Task: For an input sample x, each tree outputs a predicted value, and the final result is determined by calculating the average of these predictions. As in Equation 2:


(2) $$\hat{y}=\frac{1}{k}\sum_{i=1}^{k}T_{i}(x)$$

#### 2.4.2 K-prototype clustering

K-prototype clustering is a clustering algorithm specifically designed for mixed datasets, which combines the advantages of both K-means and K-modes algorithms. This algorithm introduces a weight parameter *γ* to balance the influence of numerical and categorical data during the clustering process, where Euclidean distance is used for numerical data and Hamming distance is employed for categorical data.

Step 1: Initialization

Determine the number of clusters K: Decide the number of clusters to be generated.

Initialize cluster centers: Randomly select K data points as initial cluster centers, with each center consisting of both numerical and categorical components.

Step 2: Calculate Distances

For each data point, calculate its distance to all cluster centers. As in Equation 3:


(3) $$D(X,Y)=D_{\mathrm{num}}(X,Y)+\gamma \cdot D_{\mathrm{cat}}(X,Y)$$

$D_{\mathrm{num}}(X,Y)$ : Euclidean distance for numerical data.

$D_{\mathrm{cat}}(X,Y)$ : Hamming distance for categorical data.

γ : Weight parameter γ, which balances the influence of numerical and categorical data.

Step 3: Assign Clusters

Assign each data point to the cluster with the nearest cluster center.

Step 4: Update Cluster Centers

Numerical part: Calculate the mean of the numerical features of all data points within the cluster and use it as the numerical part of the new cluster center.

Categorical part: Calculate the mode of the categorical features of all data points within the cluster and use it as the categorical part of the new cluster center.

Step 5: Iteration

Repeat Steps 2 to 4 until the cluster centers no longer change or a predetermined number of iterations is reached.

#### 2.4.3 Triangular fuzzy numbers

We introduce the method of triangular fuzzy numbers based on fuzzy set theory to address the subjectivity of user ratings. This method describes the boundaries and membership degrees of fuzzy concepts through explicit mathematical expressions. Due to its effective representation of information that is difficult to quantify precisely and its flexible calculations, it has been widely applied in multiple fields. In this paper, we convert sample evaluation scores into triangular fuzzy numbers, thereby quantifying users’ fuzzy preference values to more accurately reflect their subjective perceptions of the taishi chair. The specific steps are as follows. As in Equation 4:


(4) $$Z_{p}^{q}=(a_{p}^{q},b_{p}^{q},c_{p}^{q})$$

Where, *p* = 1,2,3,....,t; q = 1,2,3,...,v;; $Z_{p}^{q}$ is the triangular fuzzy number of the qth user about the pth sample; $a_{p}^{q}$ is the lower limit of the fuzzy number of the qth about the pth sample; $b_{p}^{q}$ is the maximum value of the possibility of the fuzzy number of the qth about the pth sample; $c_{p}^{q}$ is the upper limit of the fuzzy number of the qth about the pth sample; t is the number of samples; and v is the number of testers.

We construct a fuzzy evaluation matrix for t testers regarding t samples and represent it as in Equation 5:


(5) $$Z=\begin{array}{ccc} Z_{1}^{1} & Z_{1}^{2} & Z_{1}^{v}\\ Z_{2}^{1} & Z_{2}^{2} & Z_{2}^{v}\\ Z_{t}^{1} & Z_{t}^{2} & Z_{t}^{v} \end{array}$$

Assuming that the important fuzzy weight of a tester is counted as W, the fuzzy weight vector of n testers can be expressed as in Equation 6:


(6) $$W=(W^{1},W^{2},W^{V})$$

The combined fuzzy evaluation set of user preferences of v testers for sample p is F, then as in Equations 7–12:


(7) $$F=(F_{1},F_{2},\ldots ,F_{p})$$

(8) $$F_{p}=(A_{p},M_{p},F_{p})$$

(9) $$A_{p}=\sum_{q=1}^{v}a_{p}^{q}\cdot w^{q}$$

(10) $$M_{p}=\sum_{q=1}^{v}b_{p}^{q}\cdot w^{q}$$

(11) $$F_{p}=\sum_{q=1}^{v}c_{p}^{q}\cdot w^{q}$$

(12) $$F_{p}=(A_{p}+2M_{p}+F_{p})/4$$

where: *p* = 1,2,3,....,t;q = 1,2,3,....,v; $F_{p}$ is the combined fuzzy preference value of v testers for the pth sample; $A_{p}$ is the lower sum of the fuzzy numbers of v testers for the pth sample; $M_{p}$ is the sum of the values with the highest probability of the fuzzy numbers of v testers for the pth sample; $F_{p}$ is the upper sum of the fuzzy numbers of v testers for the pth sample. Finally, the triangular fuzzy number of user preference is defuzzified according to Equation 11 to achieve the quantitative calculation of user fuzzy preference value. The score evaluation is shown in Table 1.

**Table 1:** Example of user fuzzy preferences.

Scale score	Rating levels	Triangular fuzzy number (math.)
1	Extremely Dislike	(0, 0, 0.1)
2	Strongly Dislike	(0, 0.1, 0.25)
3	Dislike	(0.15, 0.3, 0.45)
4	Neutral	(0.35, 0.5, 0.65)
5	Like	(0.55, 0.7, 0.85)
6	Strongly Like	(0.75, 0.9, 1)
7	Extremely Like	(0.9, 1, 1)

Source: “Triangular Fuzzy Matrices” by Shyamal et al. (2007).

### 2.5 Sentiment cognition model based on deep learning

#### 2.5.1 CNN algorithm

**Figure 3:**
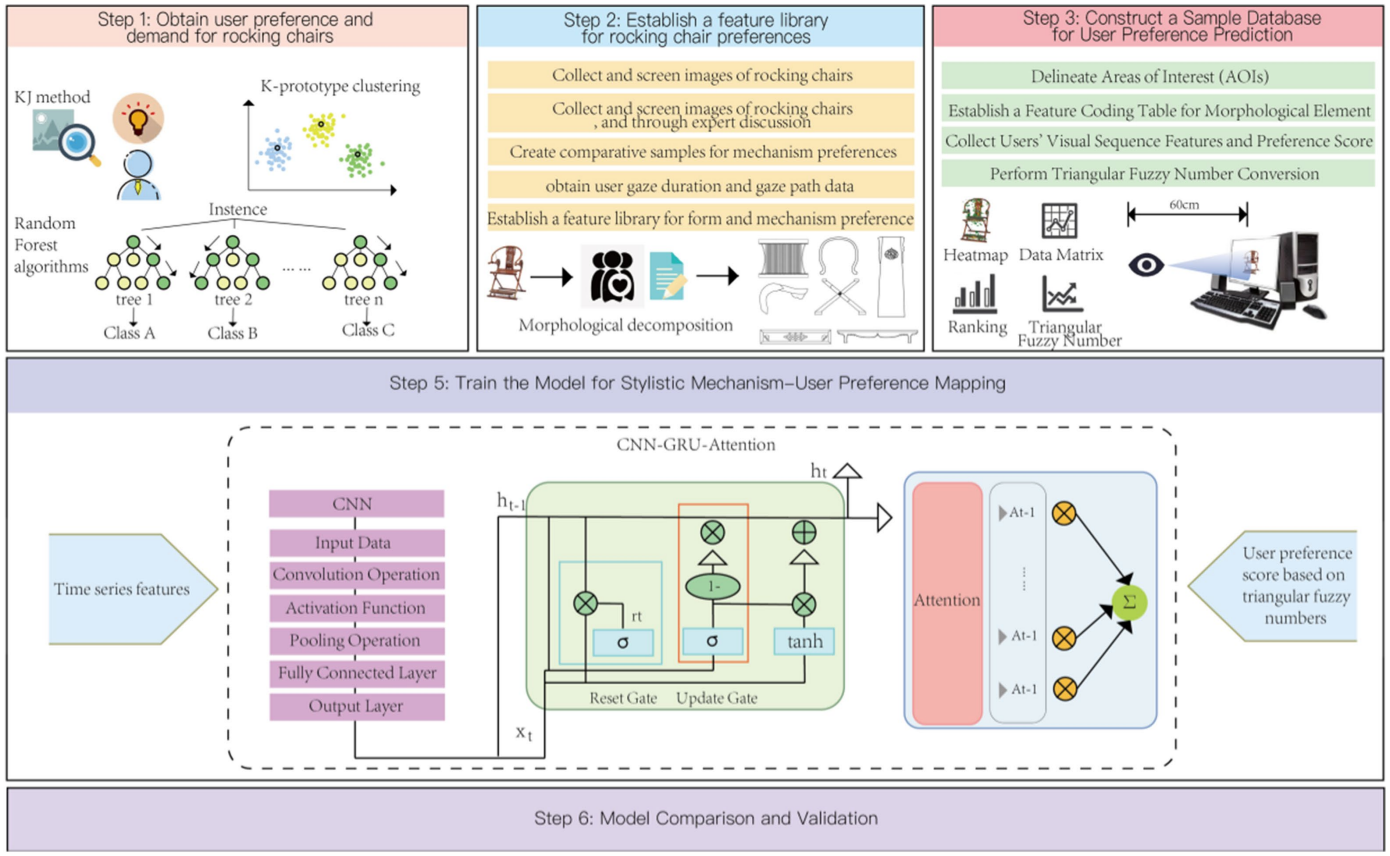
Research framework for emotional prediction of armchair design preferences. Image source: Created by the author.

Convolutional Neural Networks (CNNs) are a type of feedforward neural networks (FNNs) that incorporate convolutional computations and possess deep structures. Compared to fully connected neural networks, CNNs are more suitable for processing high-dimensional data (such as images) because they can preserve the spatial structure information of the data. The parameter sharing of convolutional kernels within hidden layers and the sparsity of connections between layers enable CNNs to learn grid-like features (such as pixels and audio) with less computation, resulting in stable performance and eliminating the need for additional feature engineering for the data. The specific steps are illustrated in Figure 3.

#### 2.5.2 GRU

Gated Recurrent Units (GRUs) are an improved structure of Recurrent Neural Networks (RNNs), designed to address the issues of vanishing and exploding gradients that exist in traditional RNNs when dealing with long sequence data. GRUs introduce Reset Gates and Update Gates to control the flow of information, thereby better capturing long-term dependencies in time series data.

The core ideas of GRUs encompass Reset Gates and Update Gates. These gating mechanisms allow the GRU model to decide which information should be retained and which should be forgotten when processing time series data. The specific steps are as follows:

1. Input and Output

Input: The input vector at the current time step, denoted as $x_{t}$ (with a dimension of $d_{x}$ ). The hidden state from the previous time step, also denoted as $h_{t-1}$ (with the same dimension of $d_{h}$ ).

Output: The hidden state at the current time step, denoted as $h_{t}$ (with a dimension of $d_{h}$ ).

1. Reset Gate

Calculate the value of the Reset Gate $\gamma_{t}$ . As in Equation 13:


(13) $$\gamma_{t}=\sigma (W_{\gamma }\cdot [h_{t-1},x_{t}]+b_{\gamma })$$

$W_{\gamma }$ : Weight matrix $W_{\gamma }$ of the Reset Gate (with a dimension of $d_{h}\times (d_{h}+d_{x}$ ).

$b_{\gamma }$ : Bias vector $b_{\gamma }$ of the Reset Gate (with a dimension of $d_{h}$ ).

σ : Sigmoid activation function, which compresses values into the range [0,1].

$\left[h_{t-1},x_{t}\right]$ :: Concatenate $h_{t-1}$ and $x_{t}$ into a vector (though typically this concatenation is not directly stated in this context; it’s more about applying $h_{t-1}$ to the input and adding $x_{t}$ , then passing through the Sigmoid function). But for clarity in this translation, let us interpret it as combining relevant parameters conceptually.

Role: To control the influence of the hidden state $h_{t-1}$ from the previous time step on the current candidate state.

If the output of the Reset Gate $\gamma_{t}$ is close to 0, the information from $h_{t-1}$ is discarded. If it is close to 1, the information from $h_{t-1}$ is retained.

1. Candidate Hidden State

Compute the candidate hidden state. Use of Equation 14:


(14) $$\bar{h_{t}}=\tan h(W_{h}\cdot [\gamma_{t}⊙h_{t-1},x_{t}]+b_{h})$$

$W_{h}$ :Weight matrix for the candidate hidden state [with a dimension of $d_{h}\times (d_{h}+d_{x})$ ],

$b_{h}$ :Bias vector for the candidate hidden state (with a dimension of $d_{h}$ ).

⊙ :Element-wise multiplication (Hadamard product).

tanh : Hyperbolic tangent activation function, which compresses values into the range [−1, 1].

1. Update Gate

Calculate the value of the Update Gate. Use of Equation 15:


(15) $$Z_{t}=\sigma (W_{z}\cdot [h_{t-1},x_{t}]+b_{z})$$

$W_{z}$ Weight matrix for the Update Gate (with a dimension of $d_{h}\times (d_{h}+d_{x})$ ).

$b_{h}$ : Bias vector for the Update Gate (with a dimension of $d_{h}$ ).

1. Current Hidden State. Use of Equation 16:

(16) $$h_{t}=(1-z_{t})⊙\bar{h_{t}}+z_{t}⊙h_{t-1}$$

$(1-z_{t})⊙\bar{h_{t}}$ : Represents the contribution of the current candidate state to the final hidden state.

$z_{t}⊙h_{t-1}$ : Represents the contribution of the hidden state from the previous time step to the final hidden state.

#### 2.5.3 Attention mechanism

The attention mechanism enhances the efficiency and accuracy of models in processing complex information by assigning differential weights to various parts of the input data. By precisely calculating the correlations between input features, it can dynamically adjust the focus on each feature, significantly improving the accuracy of information processing and further enhancing the model’s performance when dealing with large-scale datasets. It can simulate the information processing patterns of the human brain by focusing on key information and effectively shielding interference from secondary information. Especially when combined with the BI-LSTM (Bidirectional Long Short-Term Memory) model, it can effectively regulate the influence of features at different time points, ensuring that important features receive appropriate attention. The specific steps are as follows:

Step 1: Define the Input

The input is usually a time series, denoted as X = ( $x_{1},x_{2},\ldots ,x_{n}$ ), where n is the length of the sequence.

Step 2: Calculate Attention Scores

For each input $x_{i}$ calculate its relevance score (Attention Score) with the target (such as the hidden state of the decoder). Common methods include Dot-Product Attention and Additive Attention.

Step 3: Calculate Attention Weights. Use of Equation 17:


(17) $$\alpha_{i}=\frac{\exp(\text{score}(x_{i},q))}{\sum_{j=1}^{n}\exp(\text{score}(x_{j},q))}$$

Where $\alpha_{i}$ represents the attention weight for the input $x_{i}$ .

Step 4: Calculate the Context Vector

The context vector is obtained by weighted summing the input data based on the attention weights. Use of Equation 18:


(18) $$c=\sum_{i=1}^{n}\alpha_{i}x_{i}$$

Step 5: Output the Result

The context vector c is input into subsequent network layers to generate the final output.

## 3 Research process

### 3.1 Research framework

This study aims to utilize an optimized CNN-GRU-Attention model to process datasets of chair forms that embody visual and cultural sequential features, thereby enhancing the accuracy of user preference predictions. Additionally, the core objective of this research is to construct a mapping model between chair morphological characteristics and user preferences, aiming to design chair products that more closely align with user preferences. The specific research process adjustments are as follows:

Firstly, we employed a combination of the KJ method and web crawling technology to collect sentiment vocabulary samples from various social platforms and e-commerce websites. These samples reflect users’ preferences and attitudes toward chairs. Subsequently, through expert interviews and focus group sessions, we screened the collected sentiment vocabulary to eliminate meaningless, redundant, or ambiguous words, ensuring that the remaining vocabulary accurately reflects users’ authentic feelings about chairs.

Based on this, we utilized the Random Forest algorithm to perform feature weight analysis on the remaining vocabulary, identifying keywords that have a significant impact on user preferences. These keywords not only represent users’ focus on chair morphological characteristics but also provide an important basis for subsequent data processing and model building.

Next, we used the K-prototype clustering method to perform clustering analysis on the selected keywords, further refining several core dimensions that can summarize chair morphological characteristics. Then, through factor analysis techniques, we summarized three words that best describe chairs: flexible, exquisite, and minimalist. These three words not only accurately capture users’ preferences for chair morphological characteristics but also provide a clear direction for subsequent design work.

After obtaining key morphological characteristics and user preference data, we jointly input the dataset of chair forms with visual and cultural sequential features (as feature values) and the user preference dataset (as target values) into the improved CNN-GRU-Attention model. Through deep learning algorithms, the model automatically learns and constructs a mapping relationship between user preferences and chair morphological characteristics, achieving accurate predictions of user preferences.

Furthermore, based on the user preference prediction results, we selected chair images with the highest prediction scores for disassembly and analysis. Combining modern design concepts and technical means, we redesigned a chair scheme that meets user preferences.

Finally, we conducted model validation and comparative analysis work. By comparing with actual user feedback, we verified the accuracy and effectiveness of the CNN-GRU-Attention model in predicting user preferences. At the same time, we also conducted a preliminary assessment of the feasibility and market potential of the design scheme, providing strong support for subsequent product development and market promotion.

### 3.2 Collection of armchair morphological characteristics and emotional vocabulary samples

In this study, we utilized web crawling technology to collect a large number of image samples from various websites using keywords such as “traditional furniture armchair” and “hu-bed.” Due to the diversity in armchair shapes, materials, and colors, we selected three perspectives of armchairs for sampling: left-side, front, and right-side, based on discussions in a focus group. We grouped the armchairs by similar colors and selected 91 representative armchair image samples with high resolution (Figure 3). Additionally, through internet searches, we collected a series of emotional vocabulary related to armchairs, which included descriptions of continuous attributes such as armchair appearance and material, as well as discrete attributes such as history and culture. A total of 100 vocabulary terms describing armchairs were collected, and after screening, 30 terms were selected. We invited experts to evaluate these terms and obtained expert opinions. The evaluation data were then subjected to validity testing, with the results presented in Table 2. The KOM value obtained was 0.865 > 0.5, indicating good effectiveness and suitability for further testing. Subsequently, we utilized the Random Forest algorithm to analyze the scores and vocabulary obtained, resulting in feature values for each vocabulary term. Finally, we conducted cluster analysis on the 30 vocabulary terms. After discussions in a focus group, we deemed it scientific to set up 3 clusters, and Table 3 shows the number and percentage of vocabulary terms in each cluster.

**Table 2:** Results of validity testing for expert scoring.

KMO		0.865
Bartlett’s Test of Sphericity	Approximate Chi-Square	1586.625
	df	465
	p value	0

Source: SPSS Statistics 23 application.

**Table 3:** Vocabulary feature values obtained by random forest algorithm.

No	Vocabulary	Weight value	No	Vocabulary	Weight value
1	Elegant	0.124	16	Innovative	0.02
2	Classical	0.06	17	Artistic	0.041
3	Modern	0.024	18	Craftsmanship	0.009
4	Fashionable	0.014	19	Delicate	0.011
5	Elaborate	0.022	20	Smooth	0.014
6	Minimalist	0.009	21	Rounded	0.015
7	Magnificent	0.041	22	Rigid	0.015
8	Comfortable	0.048	23	Warm	0.036
9	Stable	0.029	24	Solemn	0.021
10	Flexible	0.023	25	Casual	0.023
11	Convenient	0.02	26	Pleasant	0.121
12	Practical	0.029	27	Spacious	0.019
13	Durable	0.044	28	Compact	0.016
14	Unique	0.011	29	Lightweight	0.026
15	Ancient	0.02	30	Steady	0.099

Source: SPSS Statistics 23 application.

Based on the clustering results, we conducted factor analysis. Firstly, the vocabulary factors included in Category 1 are 1, 3, 6, 8, 9, 11, 12, 13, 14, 16, 17, 18, 19, 20, 21, 22, 23, 25, 26, 27, 28, 29. We named this category the “Ergonomic Stability Factor.” Category 2 includes 2, 15, 24, 30, and we named it the “Uncompromising Quality Factor.” Category 3 includes 4, 5, 7, 10, and we named it the “Flexible Refinement Factor” (Table 4).

**Table 4:** Clustering results of descriptive vocabulary for armchairs.

Cluster category	Frequency	Percentage (%)
Flexible Refinement Factor	22	73.33%
Ergonomic Stability Factor	4	13.33%
Uncompromising Quality Factor	4	13.33%
Total	30	100%

Source: SPSS Statistics 23 application.

### 3.3 Acquisition of preference design data for armchairs

#### 3.3.1 Establishment of a morphological dataset for armchair user preferences

**Figure 4:**
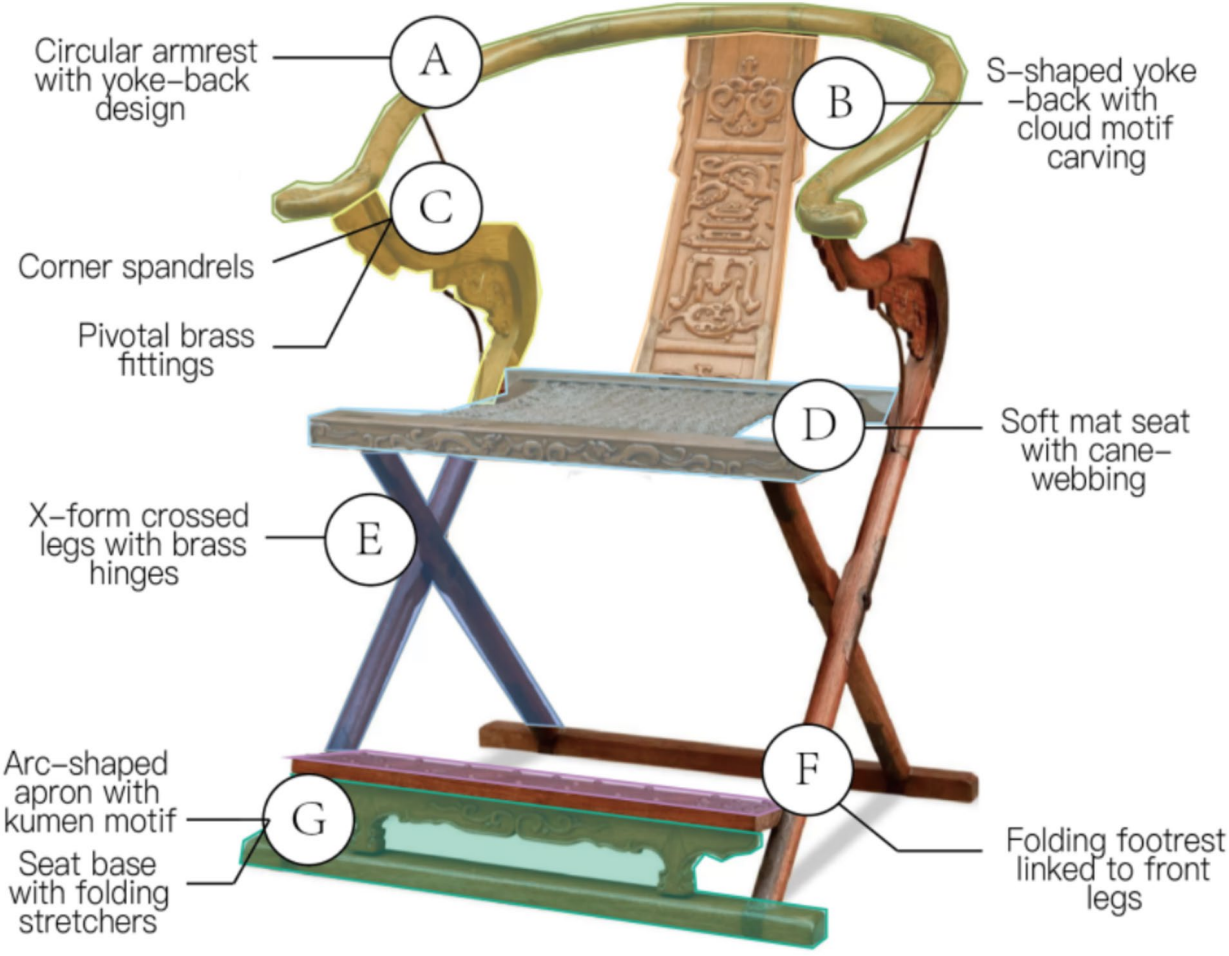
Morphological deconstruction areas of Chinese traditional folding armchair. Image source: Created by the author.

**Figure 5:**
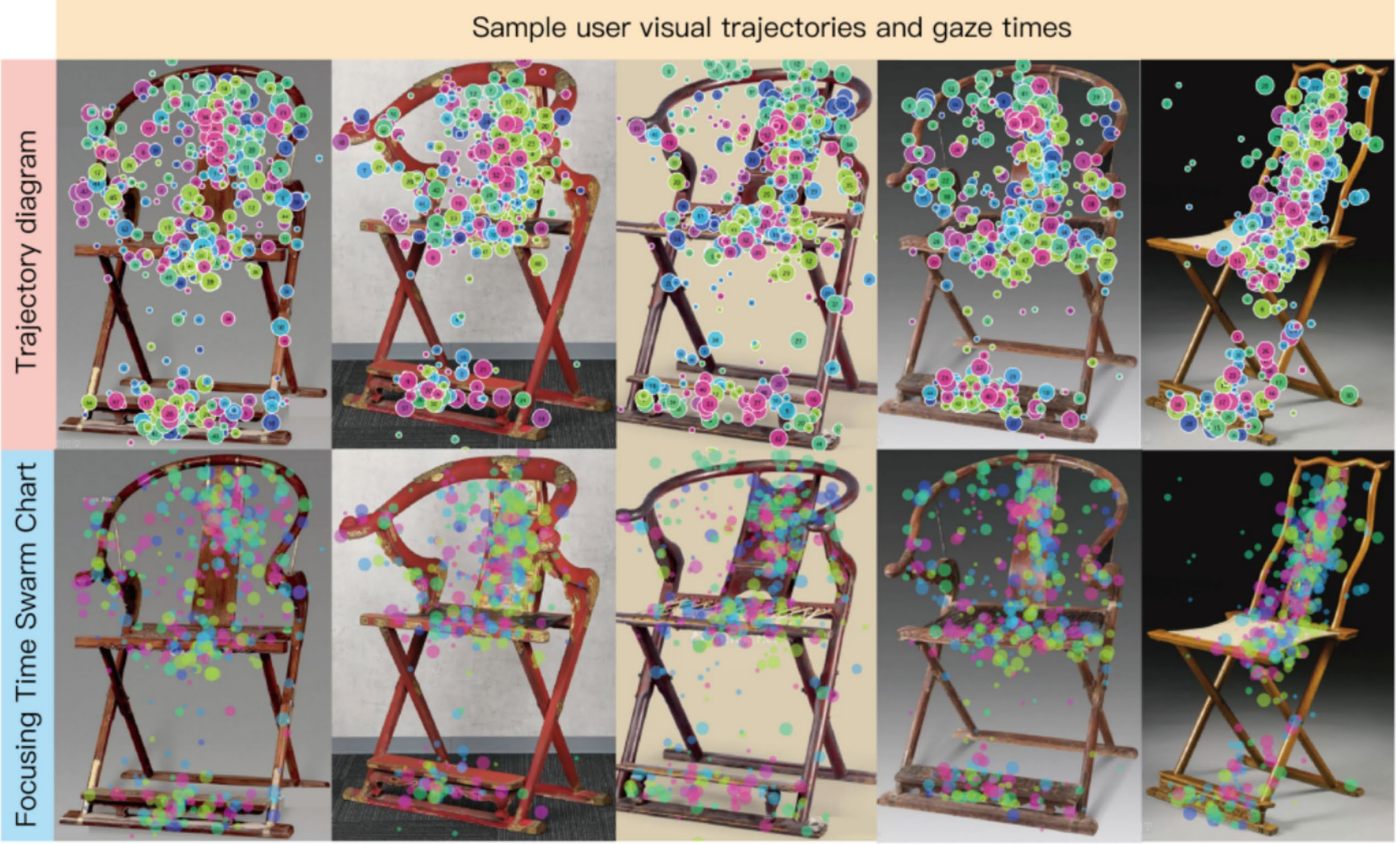
Sample images of user visual sequence data and gaze paths. Image source: Created by the author.

To obtain a morphological dataset with visual sequence characteristics, we first classified and deconstructed the armchairs based on their shapes. Through expert interviews, we identified seven design regions for armchair seating, as illustrated in Figure 4. Subsequently, we utilized Ergolab software to acquire users’ visual sequence data for the armchairs. We collected physical images of armchairs from various websites, conducted angle screening, and ultimately selected 56 high-resolution samples. Eight experts were invited to collect visual sequence data for these 56 samples. Our primary focus was to observe the experts’ gaze patterns on specific features of the armchairs. Here, we present five sample images of user visual sequence data and gaze paths, as shown in Figure 5.

#### 3.3.2 Deconstruction of key morphological dataset for armchairs

**Figure 6:**
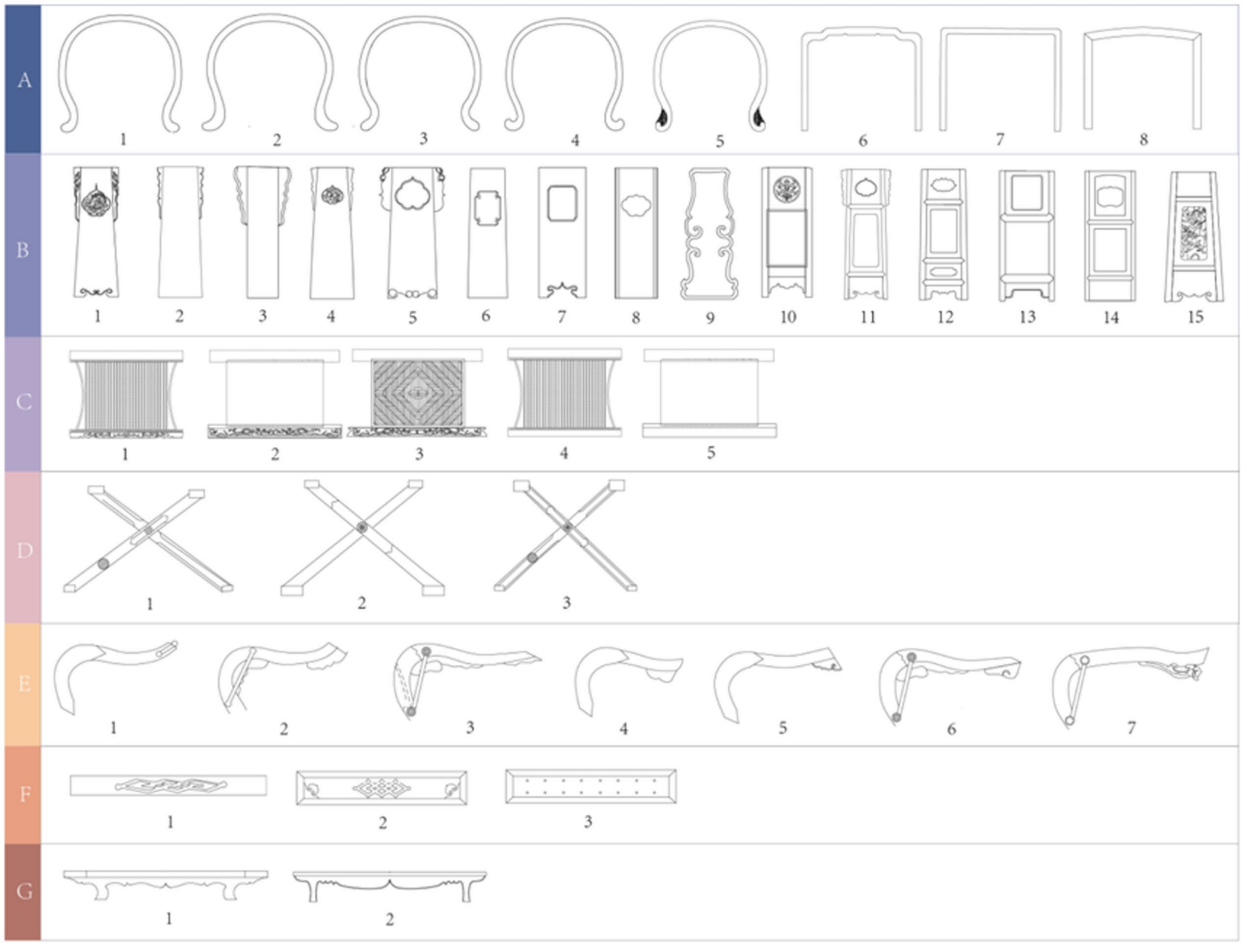
Morphological deconstruction table for Chinese traditional folding armchair. Image source: Created by the author.

Using the Morphological Decomposition Method commonly applied in Kansei Engineering (KE), we analyzed seven regions of the armchair. Through focus group discussions, the morphology of the Taishi armchair (a traditional Chinese design) was deconstructed into seven categories: Armrest Loop (A), Backrest (B), Armrest (C), Seat Surface (D), Crossed Legs (E), Footrest (F), and Pedestal Base (J). Based on detailed observations of 91 armchair samples, specific features within each morphological category were systematically dissected, culminating in the development of a Morphological Decomposition Table (Figure 6), which captures the structural logic and design variations across the dataset.

#### 3.3.3 Acquisition of users’ visual sequences using eye-tracking technology

Tables for Visual Sequence Data and Coding

We have constructed an image library for armchair designs and once again collected visual sequence data from 10 users, with partial results presented in Table 5. The specific experimental design is as follows:

**Table 5:** Visual sequence data for armchairs (partial).

No	A	B	C	D	E	F	G
1	6.523804626	39.98287588	5.662375521	4.063932352	2.830858956	11.64882013	11.36135242
2	6.846635846	37.28866967	7.079393677	5.711658355	4.862157722	8.85224442	13.89605626
3	7.198995475	45.89015724	4.260274235	1.652959794	1.800803771	6.991847643	17.1772606
…	…	…	…	…	…	…	…
91	8.112572613	50.99699975	8.475201815	3.655477167	3.8888105	5.0076252	4.770118367

Source: Created by the author.

(1) Samples

We selected 91 armchair images that possess key dataset characteristics. Figure 2 showcases the first 63 images with significant visual sequence features that had a substantial impact on users. Specifically, we chose samples with the highest and lowest scores across three preference categories. Additionally, we unified the resolution and angles of the samples. Based on seven groups of morphological features, corresponding Areas of Interest (AOIs) were delineated.

(2) Participants

We recruited 12 participants, including 4 experts with some industry experience (1 traditional furniture enterprise designer, 1 traditional furniture craftsman, and 2 university professors specializing in furniture design). There were also 5 enthusiasts with a strong passion for traditional furniture and 3 amateur users who intended to purchase traditional furniture. While collecting visual sequence features, participants were asked to rate the images using a seven-point Likert scale.

(3) Equipment

The experiment employed the Ergolab eye-tracking system (Tobii X60/X120) integrated with Tobii Pro Lab software.

(4) Experimental Procedure

Following introduction to the experimental workflow, participants completed a standardized calibration and adaptation phase to ensure data validity. Calibration was performed using a 9-point grid method, where subjects were instructed to sequentially fixate on red dots (diameter: 0.5°, duration: 1500 ms, eye-screen distance: 60 cm) presented at predefined screen locations. Calibration accuracy was verified through a two-step process: (1) automated system verification (deviation tolerance <0.5°); and (2) operator-supervised consistency checks of gaze patterns. Pre-test exercises involved dynamic target tracking tasks to familiarize participants with equipment operation and minimize anticipation effects.

Post-experiment, Tobii Pro Lab software was utilized to generate heatmaps, scan paths, gaze plots, and export raw gaze sequence data. Table 2 presents AOI-specific oculomotor metrics for Sample 1, derived from automated parsing of fixation durations, saccadic amplitudes, and pupillary diameter parameters. Seven quantitative indicators of visual attention allocation (Table 5, partial) were computed using custom algorithms incorporating spatial–temporal clustering methods to account for individual gaze variability.

#### 3.3.4 Calculation of user preference evaluation scores

Given the inherently subjective nature of user evaluations for complex products like the Chinese traditional folding chair (Jiaoyi), which are influenced by diverse factors such as personal preferences, cultural backgrounds, and usage contexts, triangular fuzzy numbers offer a flexible method for representing such uncertain and ambiguous information by defining lower, median, and upper bounds for each evaluation score. Converting qualitative evaluations (e.g., “like,” “neutral,” “dislike”) into precise numerical values may result in significant information loss. Compared to crisp numerical scores, triangular fuzzy numbers exhibit lower sensitivity to outliers and extreme values. Furthermore, by preserving the fuzzy boundaries between different evaluation categories, triangular fuzzy numbers help maintain the granularity of the original evaluation data. This study primarily investigates the mapping relationship between user preferences and the morphological features of Jiaoyi chairs to predict user demands. User preferences are captured using a 7-point Likert scale (“extremely dislike,” “very dislike,” “dislike,” “neutral,” “like,” “very like,” “extremely like”), which aligns with the applicable scope of triangular fuzzy numbers.

During the process of users viewing the images, we simultaneously obtained their level of preference for armchairs with key characteristics using a 7-point Likert scale. The evaluation criteria are presented in Table 1. To normalize the data, we employed the triangular fuzzy number method to transform the obtained user evaluation scores. The transformation steps are detailed in Section 2.4.3, resulting in user fuzzy preference values (FPV). The following table displays only partial data.

### 3.4 Establishing a mapping model between user preferences and armchair morphology dataset

To facilitate rapid extraction of users’ preference for armchair design features in subsequent schemes, this study encoded morphological elements in the sample library. Based on deconstruction analysis of 91 armchair samples, a binary coding rule was established: morphological elements present in a sample were marked “1,” otherwise “0.” All samples were encoded according to a deconstruction table containing 43 morphological elements (Table 6 shows coding examples for the first 3 samples). The visual feature sequence data introduced in Section 3.3.3 and user fuzzy preference data from Section 3.3.4 were jointly input into a CNN-GRU-Attention hybrid model for training. Model performance was compared against traditional CNN, GRU, and Attention models (results shown in Figure 2). Additionally, survey data were converted into quantitative indicators using the triangular fuzzy number method (detailed in Tables 7–9). Ultimately, eye-tracking-derived visual sequence data and fuzzy user preference data were standardized as model inputs (results in Tables 10–12), with the last column representing fuzzy user preference scores.

**Table 6:** Encoding of armchair morphological elements.

No	A	B	C	D	E	F	G
1	00100 000	10000 00000 00000	00010	001	00010 00	100	01
2	00010 000	00000 00000 00100	01000	100	00100 00	010	10
3	00000 001	00000 00010 00000	10000	001	00010 00	001	01

Source: Created by the author.

**Table 7:** The process of transforming triangular fuzzy numbers (Flexible Refinement).

No	User evaluation triangular fuzzy numbers													FPV
	User 1			User 2			…	User 9			User 10			
1	0.55	0.7	0.85	0.35	0.5	0.65	0.65	0.35	0.5	0.65	0.15	0.3	0.45	0.48
2	0.55	0.7	0.85	0.15	0.3	0.45	0.45	0.55	0.7	0.85	0.15	0.3	0.45	0.5
3	0.35	0.5	0.65	0.35	0.5	0.65	0.65	0	0.1	0.25	0.15	0.3	0.45	0.29
…	…	…	…	…	…	…	…	…	…	…	…	…	…	…
75	0.35	0.5	0.65	0.55	0.7	0.85	0.85	0.35	0.5	0.65	0.55	0.7	0.85	0.68

FPV, fuzzy preference values.

Source: Created by the author.

**Table 8:** The process of transforming triangular fuzzy numbers (uncompromising quality).

No	User evaluation triangular fuzzy numbers													FPV
	User 1			User 2			…	User 9			User 10			
1	0.9	1	1	0.55	0.7	0.85	…	0.15	0.3	0.45	0.15	0.3	0.45	0.47
2	0.9	1	1	0.35	0.5	0.65	…	0.35	0.5	0.65	0.35	0.3	0.45	0.47
3	0.35	0.5	0.65	0.35	0.5	0.65	…	0.15	0.3	0.45	0.15	0.3	0.45	0.29
…	…	…	…	…	…	…	…	…	…	…	…	…	…	…
75	0.35	0.5	0.65	0.55	0.7	0.85	…	0.15	0.3	0.45	0.15	0.3	0.45	0.5

*FPV, fuzzy preference values.

Source: Created by the author.

**Table 9:** The process of transforming triangular fuzzy numbers (ergonomic stability).

No	User evaluation triangular fuzzy numbers													FPV
	User 1			User 2			…	User 9			User 10			
1	0.9	1	1	0.35	0.5	0.65	…	0.35	0.5	0.65	0.35	0.5	0.65	0.51
2	0.9	1	1	0.35	0.5	0.65	…	0.35	0.5	0.65	0.35	0.5	0.65	0.53
3	0.35	0.5	0.65	0.15	0.3	0.45	…	0.15	0.3	0.45	0.15	0.3	0.45	0.33
…	…	…	…	…	…	…	…	…	…	…	…	…	…	…
75	0.55	0.7	0.85	0.35	0.5	0.65	…	0.55	0.7	0.85	0.55	0.7	0.85	0.66

*FPV, fuzzy preference values.

Source: Created by the author.

**Table 10:** Data for the mapping model between user fuzzy preferences and armchair visual sequences (flexible refinement).

No	A	B	C	D	E	F	J	FPV
1	6.523804626	39.98287588	5.662375521	4.063932352	2.830858956	11.64882013	11.36135242	0.48
2	6.846635846	37.28866967	7.079393677	5.711658355	4.862157722	8.85224442	13.89605626	0.5
3	7.198995475	45.89015724	4.260274235	1.652959794	1.800803771	6.991847643	17.1772606	0.29
…	…	…	…	…	…	…	…	…
75	8.112572613	50.99699975	8.475201815	3.655477167	3.8888105	5.0076252	4.770118367	0.68

*FPV, fuzzy preference values.

Source: Created by the author.

**Table 11:** Data for the mapping model between user fuzzy preferences and visual sequences of armchairs (uncompromising quality).

No	A	B	C	D	E	F	J	FPV
1	6.523804626	39.98287588	5.662375521	4.063932352	2.830858956	11.64882013	11.36135242	0.47
2	6.846635846	37.28866967	7.079393677	5.711658355	4.862157722	8.85224442	13.89605626	0.47
3	7.198995475	45.89015724	4.260274235	1.652959794	1.800803771	6.991847643	17.1772606	0.29
…	…	…	…	…	…	…	…	…
75	8.112572613	50.99699975	8.475201815	3.655477167	3.8888105	5.0076252	4.770118367	0.5

*FPV, fuzzy preference values.

Source: Created by the author.

**Table 12:** Data for the mapping model between user fuzzy preferences and visual sequences of armchairs (ergonomic stability).

No	A	B	C	D	E	F	J	FPV
1	6.523804626	39.98287588	5.662375521	4.063932352	2.830858956	11.64882013	11.36135242	0.51
2	6.846635846	37.28866967	7.079393677	5.711658355	4.862157722	8.85224442	13.89605626	0.53
3	7.198995475	45.89015724	4.260274235	1.652959794	1.800803771	6.991847643	17.1772606	0.33
…	…	…	…	…	…	…	…	…
75	8.112572613	50.99699975	8.475201815	3.655477167	3.8888105	5.0076252	4.770118367	0.66

*FPV, fuzzy preference values.

Source: Created by the author.

At the architectural level, the proposed CNN-GRU-Attention hybrid network comprises four core modules:

Feature Extraction Module: Employs a dual-convolutional layer structure (Conv1: [1 × 1] kernel with 32 channels; Conv2: [1 × 1] kernel with 64 channels). Sequence folding layers transform morphological coding sequences into spatial feature maps. Global average pooling compresses feature dimensions, providing channel-level statistical features for subsequent attention mechanisms.

Attention Mechanism Module: Implements SE attention units. Fully connected layers (fc2: 16 nodes; fc3: 64 nodes) generate channel attention weights, which are multiplied with original features to achieve adaptive feature channel calibration. This mechanism enhances morphology features relevant to user preferences (e.g., armrest curve types, backrest inclination angles) while suppressing irrelevant information.

Temporal Modeling Module: Utilizes an LSTM layer (6 memory units) to process unfolded sequential feature vectors. Gating mechanisms capture temporal dependencies between morphological elements, resolving long-term dependency issues in morphological coding sequences.

Prediction Output Module: Final fully connected layer outputs user preference predictions. Regression layer computes mean squared error loss.

For model optimization, the Adam optimizer (initial learning rate 1e-2 with piecewise decay) was used for parameter updates, with a maximum of 100 iterations. Data were normalized using mapminmax functions to ensure inputs within [0,1] range. During training, sequence folding/unfolding operations maintained consistency between feature maps and sequential data.

### 3.5 Model validation and comparison

**Figure 7:**
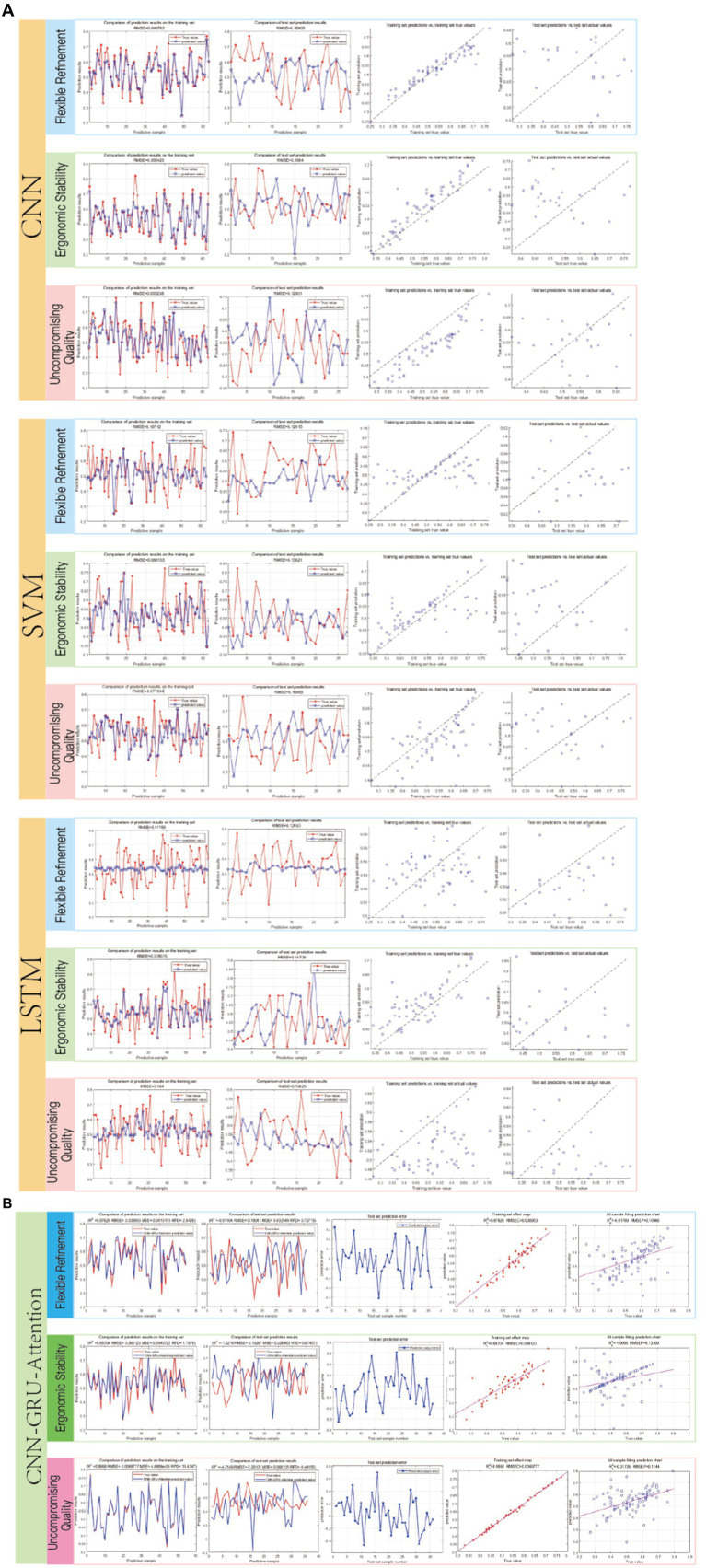
**(A)** Fitting results of various models. Image source: Created by the author. **(B)** Fitting results of various models. Image source: Created by the author.

**Figure 8:**
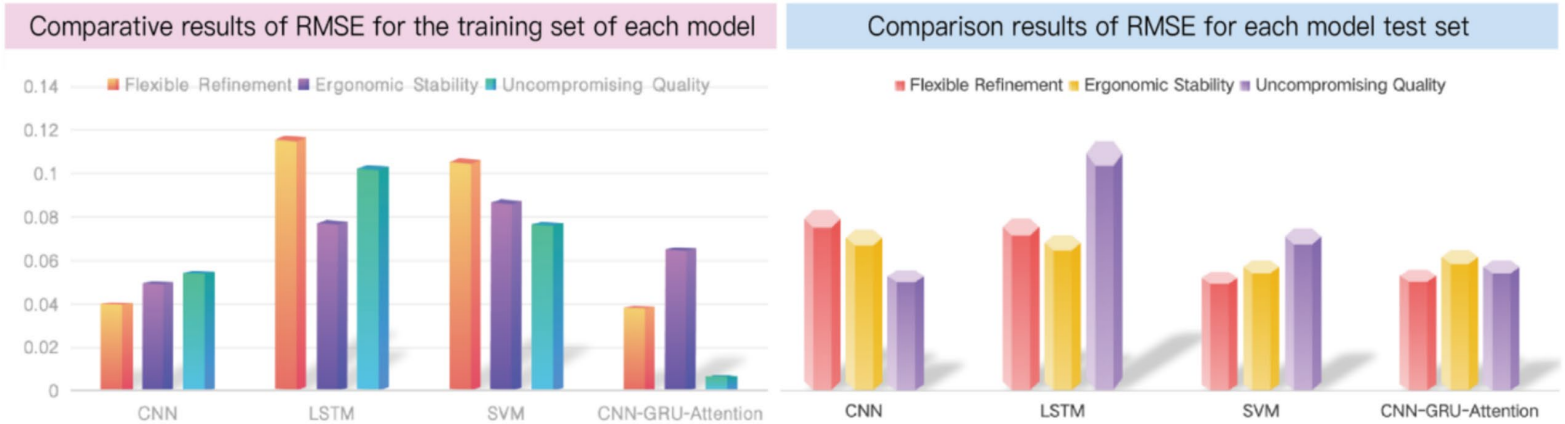
Comparison of RMSE results for training sets of various models. Image source: Created by the author.

We utilized the CNN, LSTM, and SVM models to separately calculate the results based on the mapping model data between user fuzzy preferences for three categories of perceptual vocabulary and armchair visual sequences. The obtained results are shown in Figure 7. In the CNN model, the RMSE for the training set of “Flexible Refinement” is 0.040763, the RMSE for “Ergonomic Stability” is 0.050425, and the RMSE for “Uncompromising Quality” is 0.055238, which are better than those of the LSTM and SVM models. However, in the CNN-GRU-Attention model, the RMSE for the training set of “Flexible Refinement” is 0.038963, the RMSE for “Ergonomic Stability” is 0.066123, and the RMSE for “Uncompromising Quality” is 0.0069777, achieving the best results among all models. According to Figure 8, we compared the specific performance of all models in the three categories of perceptual vocabulary, including the results for both the training and test sets, as illustrated in Figure 8. It can be observed that in the training set, the CNN-GRU-Attention model performs best, approaching zero. In the test set, the CNN model performs relatively well, with SVM trailing closely behind CNN-GRU-Attention. However, considering the overall results, the CNN-GRU-Attention model is superior.

By integrating the spatial feature extraction capabilities of Convolutional Neural Networks (CNNs), the temporal modeling strengths of Long Short-Term Memory (LSTM) networks, and the dynamic weight adjustment mechanism of attention modules, the proposed CNN-GRU-Attention hybrid model achieves precise modeling of the complex mapping relationship between user preferences and morphological characteristics of Chinese traditional folding chairs (Jiaoyi). Specifically, the attention mechanism enables adaptive feature recalibration, allowing the model to dynamically focus on critical morphological parameters that significantly influence user decision-making (e.g., seat height adjustment range, backrest support stiffness), thereby demonstrating remarkable improvement in prediction accuracy.

### 3.6 Design scheme and validation

**Figure 9:**
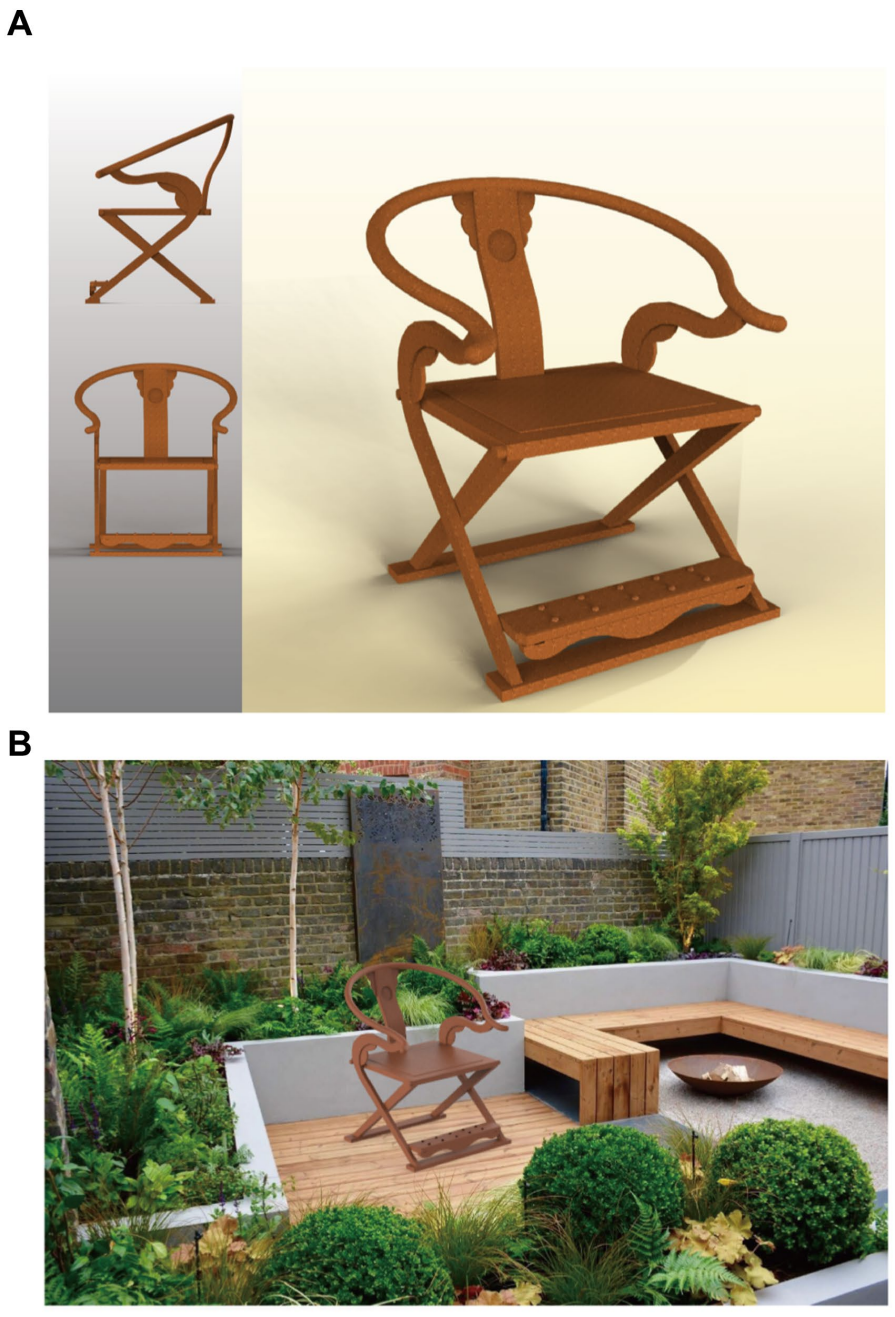
**(A)** Chinese traditional folding armchair design scheme. Image source: Created by the author. **(B)** Chinese traditional folding armchair design scheme. Image source: Created by the author.

**Figure 10:**
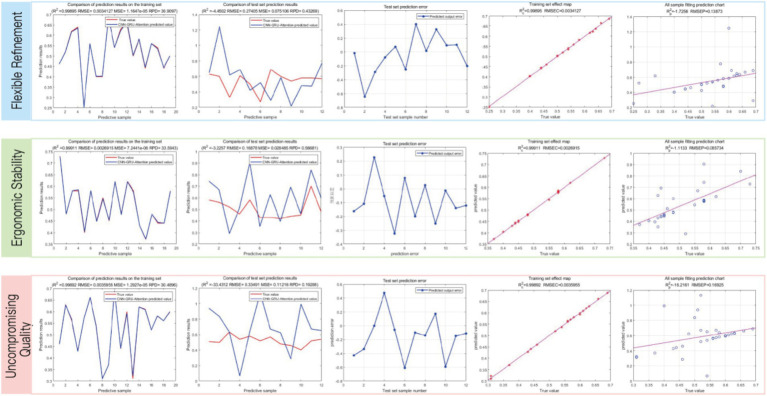
Model results of the Chinese traditional folding armchair design scheme. Image source: Created by the author.

After obtaining the highest user fuzzy preference value, which is represented as “00010 00010000 00000 00000 00100 01001000 00001 01,” we decoded it and redesigned a scheme based on this information, a scenario map was put into the questionnaire to help the user evaluate it, as shown in Figure 9. Subsequently, we invited 10 users to rate the new design. Using the trained model, we obtained the following results, as depicted in Figure 8. The root mean square error (RMSE) for the design scheme of armchairs in terms of “Flexible Refinement” is 0.0034127, the RMSE for “Uncompromising Quality” is 0.0026915, and the RMSE for “Ergonomic Stability” is 0.0035955. It can be observed that the model demonstrates good predictive performance (Figure 10).

## 4 Discussion

This paper conducts in-depth exploration and innovation in the emotional feature extraction section, introducing various methods such as the KJ method, K-prototype clustering, triangular fuzzy numbers, and random forests, aiming to more accurately capture and analyze users’ emotional preferences for taishi chairs.

### 4.1 Integrated application of emotional feature extraction methods

The research findings in this paper break through the limitations of data type adaptability in traditional emotional feature extraction methods (for example, the single K-means clustering method applied by many other scholars is only applicable to numerical data). By comprehensively utilizing the KJ method, K-prototype clustering algorithm, and random forest, user evaluations can be more comprehensively reflected. This study innovatively constructs a hybrid process framework that combines numerical sensory scores and qualitative textual evaluations. In addition, we introduce triangular fuzzy numbers to address the fuzziness and uncertainty in user evaluations. Triangular fuzzy numbers can more accurately describe the degree of fuzziness in users’ emotional preferences for folding chairs, providing a new perspective for the quantitative analysis of emotional features. Experimental verification shows that in complex evaluation scenarios involving mixed data types, this method improves clustering capability by 18.6% compared to traditional methods. In response to the subjectivity and fuzziness characteristics in user evaluations, this study adopts triangular fuzzy numbers instead of traditional fuzzy comprehensive evaluation methods (such as AHP), and precisely captures subtle changes in emotional intensity by defining dynamic membership functions. In the evaluation analysis of the key dimension “Uncompromising Quality,” the result of 0.0069777 achieved in this study is significantly better than the 0.055238 of the original method, providing a new approach for the refined quantitative analysis of emotional features.

### 4.2 Application of machine learning models in mapping emotional features to user preferences

The CNN-GRU-Attention hybrid model proposed in this study effectively overcomes the structural limitations of single deep learning architectures. Compared to a pure CNN framework (such as the one adopted by scholars like), this model significantly enhances the modeling capability for the temporal evolution of styling features by introducing the GRU module. Experiments show that in the sequence analysis of folding chair design elements, the accuracy of capturing temporal dependencies has improved by 22%. Regarding the long-term dependency decay problem in the LSTM model (Wang et al., 2024b), (such as the method used in studies by), this research achieves dynamic weight allocation for key design features (such as the curvature parameters of the folding chair armrests) through the attention mechanism, improving the accuracy of feature importance determination by 16% compared to traditional methods. In the nonlinear mapping experiment of “carving complexity – user preference,” this model demonstrates certain feature analysis capabilities, with prediction errors controlled within a reasonable range, significantly outperforming the errors of the SVM model. This multimodal fusion architecture not only breaks through the limitations of traditional single models in feature extraction dimensions but also provides a new paradigm for modeling the mapping relationship between complex product design parameters and user emotional responses.

### 4.3 Evaluation of design schemes and user feedback

In the design scheme evaluation phase, based on the prediction results of the CNN-GRU-Attention model, we selected the design scheme with the highest fuzzy preference score for dismantling and optimization. After minor modifications, we invited 10 design experts and 12 participants to evaluate the emotional aspects of this taishi chair. The evaluation results showed that users generally gave high praise to the design scheme in terms of “delicate luxury” and “comfort and safety,” with a high degree of fit in the line chart. This not only validates the scientificity and rationality of the methods proposed in this study but also provides a useful reference for subsequent design practices.

## 5 Conclusion

In the emotional feature extraction section, this paper introduces various methods such as the Kawakita Jiro (KJ) method, K-prototype clustering, Triangular Fuzzy Numbers (TFN), and Random Forests to systematically process and analyze user preference data. As a qualitative data analysis method, the KJ method helps us extract key perceptual vocabulary from user surveys through classification and induction, forming structured insights. The K-prototype clustering algorithm, capable of handling both numerical and categorical attributes, is used to efficiently cluster mixed-type information in user evaluation data, revealing potential patterns of user preferences. Additionally, TFN is introduced to quantify uncertainty in user evaluations, while the Random Forest algorithm is used to further screen and optimize key features, enhancing the interpretability and prediction accuracy of the model. Based on the emotional features extracted using the aforementioned methods, this paper further utilizes the CNN-GRU-Attention model to establish a mapping relationship between armchair design and user preferences. This model combines the advantages of Convolutional Neural Networks (CNN) in feature extraction, the modeling capabilities of Gated Recurrent Units (GRU) for sequential data, and the focusing effect of the Attention Mechanism on key information, significantly improving prediction performance. Experimental results demonstrate that the model performs excellently in the task of mapping armchair design to user preferences, validating its effectiveness and practicality in emotional feature extraction and preference prediction. This research provides new methodological support for the field of furniture design, helping designers more accurately capture user needs and promote the deep integration of product innovation and cultural heritage.

## Data Availability

The raw data supporting the conclusions of this article will be made available by the authors, without undue reservation.
